# A Design Approach to Optimise Secure Remote Three-Dimensional (3D) Printing: A Proof-of-Concept Study towards Advancement in Telemedicine

**DOI:** 10.3390/healthcare10061114

**Published:** 2022-06-15

**Authors:** Xiao Wen Kok, Anisha Singh, Bahijja Tolulope Raimi-Abraham

**Affiliations:** 1 Institute of Pharmaceutical Science, King’s College London, School of Cancer and Pharmaceutical Sciences, London SE1 9NH, UK; xwkok0630@gmail.com; 2 Institute for Security Science and Technology (ISST), Imperial Business School, Imperial College London, London W12 7TA, UK; anisha@wippit.net

**Keywords:** 3D printing, additive manufacturing, telemedicine, patient-centric dosage form

## Abstract

Telemedicine is defined as the delivery of healthcare services at a distance using electronic means. The incorporation of 3D printing in the telemedicine cycle could result in pharmacists designing and manufacturing personalised medicines based on the electronic prescription received. Even with the advantages of telemedicine, numerous barriers to the uptake hinder the wider uptake. Of particular concern is the cyber risk associated with the remote digital transfer of the computer-aided design (CAD) file (acting as the electronic prescription) to the 3D printer and the reproducibility of the resultant printed medicinal products. This proof-of-concept study aimed to explore the application of secure remote 3D printing of model solid dosage forms using the patented technology, DEFEND3D, which is designed to enhance cybersecurity and intellectual property (IP) protection. The size, shape, and colour of the remote 3D-printed model medicinal products were also evaluated to ensure the end-product quality was user-focused. Thermoplastic polyurethane (TPU) and poly(lactic) acid (PLA) were chosen as model polymers due to their flexibility in preventing breakage printing and ease of printing with fused deposition modelling (FDM). Our work confirmed the potential of secure remote 3D (FDM) printing of prototype solid dosage forms resulting in products with good reproducibility, resolution, and quality towards advancements in telemedicine and digital pharmacies. The limitation of the work presented here was the use of model polymers and not pharmaceutically relevant polymers. Further work could be conducted using the same designs chosen in this study with pharmaceutically relevant polymers used in hot-melt extrusion (HME) with shown suitability for FDM 3D printing. However, it should be noted that any challenges that may occur with pharmaceutically relevant polymers are likely to be related to the polymer’s printability and printer choice as opposed to the ability of the CAD file to be transferred to the printer remotely.

## 1. Introduction

Telemedicine is defined as the delivery of healthcare services at a distance using electronic means [1]. As a result, telemedicine makes it easier for patients to receive healthcare services remotely, expanding the potential delivery of healthcare to patients across the world [2]. The telemedicine care cycle starts with healthcare providers conducting virtual medical consultations and remote diagnoses with patients using electronic means; electronic prescriptions are then produced and sent remotely to the pharmacies. It is thought that the introduction of three-dimensional (3D) printing (i.e., additive manufacturing method, where the object to be printed is developed through a computer-aided design (CAD)) in the telemedicine care cycle will transform compounding pharmacies into digital pharmacies [2,3] Moreover, 3D printing in pharmaceutical sciences allows for greater flexibility of fabrication capability in manufacturing patient-centric personalised medicines [1,4]. The incorporation of 3D printing in telemedicine could result in pharmacists designing and manufacturing personalised medicines based on the electronic prescriptions received [2]. The customised medicine would then be 3D-printed on-demand in a pharmacy setting [4]. Of concern in this area is the risk to the intellectual property (IP) during the storage, transmission, and execution of 3D printing through digital networks and systems [5,6]. Currently, in 3D printing, the entire digital file is transferred to the manufacturing device, making the digital IP vulnerable to cyberattacks, manipulation, and even theft [7]. Various solutions have been proposed to try and solve the issue of IP exposure, including blockchain, encryption, and licensing business models [8]. However, these solutions still require the complete transfer of the digital file.

The COVID-19 pandemic globally has overwhelmed health systems [9] and telemedicine has been thrust into the spotlight in the fight against COVID-19. The telemedicine approach has been employed in many different ways to better tackle the healthcare challenges that have arisen [10]. Telemedicine will likely have a more permanent place in traditional healthcare delivery long after the COVID-19 pandemic as users and providers recognise its advantages in improving global access to healthcare [9,10,11]. Even with the advantages of telemedicine, numerous barriers to uptake, such as education, cost, internet access, and patient digital literature, hinder its wider uptake [10]. Of particular concern is the cyber risk associated with the remote digital transfer of the CAD acting as the electronic prescription to the 3D printer and the reproducibility of the resultant printed medicinal products [7]. Additionally, as the shift toward telemedicine increases over time, new issues and risks as they relate to information security and privacy will need to be addressed and sufficiently managed [7].

The work presented in this proof-of-concept study aimed to explore the application of secure remote 3D printing of model solid dosage forms using the patented technology, DEFEND3D. The DEFEND3D platform is a patented secure streaming transfer protocol (SSTP), virtual inventory communications interface (VICI) designed to enhance cybersecurity and intellectual property (IP) protection. The VICI removes the need for file transfer and allows for secure digital resupply of reproduction parts remotely. An additional advantage of this technology means manufacturing and printing on demand can occur without the need for a specialist at the manufacturing or printing site [12]. The resultant remotely 3D-printed products are guaranteed to come out as designed [13].

Here, we focused on the secure remote printability of simple and complex pharmaceutically relevant designs, with a focus on evaluating their properties as they related to the patient experience when taking and accepting medication [14], i.e., visual and physical perception and optimisation of the CAD file and printing parameters (namely layer height and infill density). The size, shape, and colour of the remotely-3D-printed model medicinal products were also evaluated to ensure the end-product quality was user-focused [15]. Thermoplastic polyurethane (TPU) and poly(lactic) acid (PLA) were chosen as model polymers due to their flexibility in preventing breakage printing [16,17,18] and ease of printing with fused deposition modelling (FDM) [17,19] This proof-of-concept study seeks to explore the considerations in secure remote 3D printing towards optimisation for pharmaceutical use in the advancement of telemedicine and digital pharmacies.

## 2. Materials and Methods

### 2.1. Materials

Pink-, blue-, yellow-, and white-coloured 1.75 mm diameter thermoplastic polyurethane (TPU) filaments were purchased from Prima Creator (Malmo, Sweden) and neon pink, blue, and white 1.75 mm diameter poly(lactic) acid (PLA) filaments were purchased from Prima Creator.

### 2.2. Methods

#### 2.2.1. Computer-Aided Design(s) (CAD)

**Figure 1:**
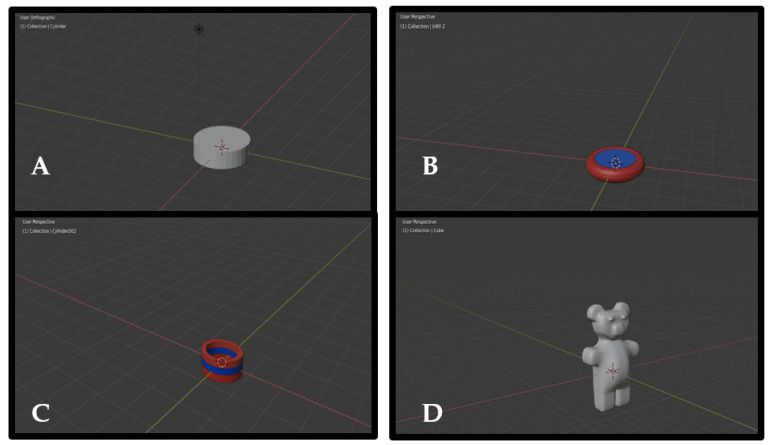
CAD of (**A**) design 1—disc shape (12 × 5 × 4 mm) (**B**) design 2—torus shape (12 × 6 × 3 mm) (**C**) design 3—ring shape (9 × 5 × 5 mm) (**D**) design 4—gummy-bear shape (11 × 20 × 3 mm), all rendered in Blender v. 2.8.

The geometry of the 3D models to be remotely printed was designed using CAD drawing software, Blender v. 2.80 (Blender Foundation, Amsterdam, The Netherlands). The designs were developed by inserting default shapes and modifying them as required. The four designs (shown in Figure 1) were selected based on work by Goyanes et al. [20] where they investigated patient acceptability of 3D-printed medicines. Their findings showed that disc (design 1), torus (design 2), ring (design 3), and gummy-bear shapes (design 4) were among the most acceptable dosage forms by patients. In our work, designs 2 and 3 represented fixed-dose combination(s) (FDC), defined as two or more drugs combined in a fixed ratio into a single dosage form [21,22]. Innovative geometries [23,24] (i.e., design 4) were also included due to their potential to improve patient compliance. Designs were also chosen for their increasing design and printing complexities to challenge the capacity of the DEFEND3d platform in ensuring the integrity of the CAD design, file, and resultant remote printability.

#### 2.2.2. Remote-Fused Deposition Modelling (FDM) 3D Printing

All designs were remotely 3D-printed using the DEFEND3D platform. Due to the secure nature of the software, the in-depth workings of the algorithm are not able to be published. However, an overview of the workings of the platform can be provided. In brief, the DEFEND3D cybersecurity and transmission protocol allows for a safe remote method for controlled reproduction of an item that is represented by a digital asset stored in a trusted computing environment using a reproduction device (i.e., a 3D printer) in an untrusted computing environment. In practical terms, this is achieved by a continuous secure stream of production instructions to the machine with the use of Microsoft Azure Cloud services. The reproduction instructions are secured by six levels of security with encryption being only one of them. Variables, such as machine type, settings, and the material used, can be preset to enforce high manufacturing standards in the production process. The DEFEND3D platform allows for CAD files to be sliced using pre-defined gcode file pre-printing [14] and, therefore, eliminating the need for complex slicing software for the final CAD file and poor quality of the resultant printed product [25,26].

This study used FDM 3D printing. FDM 3D printing is an extrusion and thermo-based 3D printing technique where thermoplastic polymers are melted at a high temperature and solidified immediately onto the previous layer on the build plate [1,3,22]. Five different printing phases were required, which showed optimisation (namely printing quality and reproducibility to original CAD design) of the model dosage forms. Model solid dosage forms were remoted and printed using a Flashforge Creator Pro Dual Extruder via the DEFEND3D platform. The polymers used, the printer extruder type and the printing parameters are provided in Table 1.

#### 2.2.3. Determination of Physical Properties

Visual observation, the physical properties, namely weight, diameter (d), length (l), and thickness (t) surface area (two dimensional (2D) and theoretical), and volume of the model solid dosage forms were recorded. A computerised surface analysis using ImageJ software (Bethesda, Maryland, USA) to calculate the two-dimensional (2D) surface area of printed products was carried out. The scale was calibrated to 300 distance in pixels of a known distance of 1, where the scale was set as 300 pixels/mm. The theoretical surface area (SA) and volume (Vol) were calculated using the equations listed in Table 2.

## 3. Results

**Figure 2:**
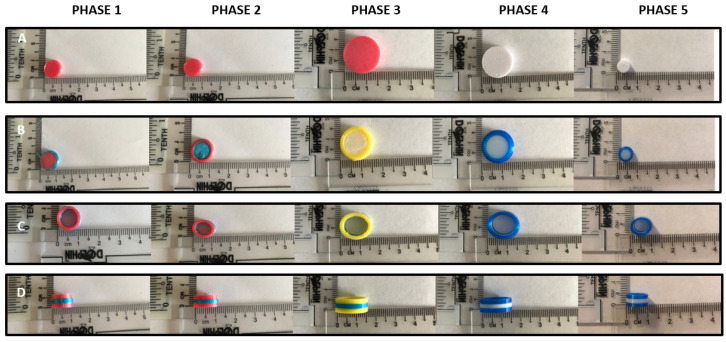
The 2D images of remotely-3D-printed model designs (**A**) 1, (**B**) 2, (**C**) 3—top view and (**D**) 3—side view phases 1 to 5.

**Figure 3:**
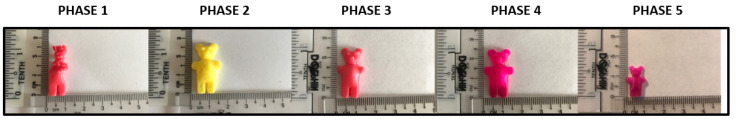
The 2D images of remotely 3D-printed design 4, phases 1 to 5.

**Figure 4:**
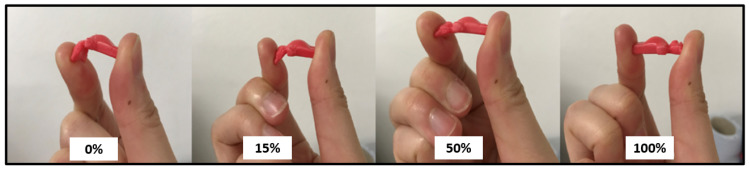
Design 4 (i.e., gummy bear shape) remotely 3D-printed at 0%, 15%, 50%, and 100% infill densities, showing different degrees that the prototypes could be bent manually (as an indication of flexibility).

The design 1 to 5 prototypes were successfully remotely 3D-printed via the DEFEND3D platform using TPU and PLA printing filaments. Evaluations of the resultant remotely-printed products (general printability, visual appearance, and physical properties) are shown per design (Figure 2, Figure 3 and Figure 4 and Table 3, Table 4 and Table 5).

### 3.1. Remote Printability

All designs were remotely printable with varied resolutions, which were optimised with changes in the printing parameters (namely base speed, layer height, infill density, and extrusion temperature) and are shown in Figure 2A–D. Prototypes remotely printed in printing phases 1 to 3 were modified to optimise geometry. The phase 1 model solid dosage form prototypes were of clinical relevance; they were designed to be within the range of size 2 (18 × 6.35 mm) and size 3 capsules (15.9 × 5.82 mm) [14,15,20,27]. Printing phases 4 and 5 involved modifications to optimise the resolution and quality of the remotely-printed model solid dosage forms.

### 3.2. Visual Observations and Physical Properties of Remotely-Printed Products for Designs 1 to 3

The remotely-3D-printed design 1 (disc shape), shown in Figure 2A, was the simplest design to be printed in this study. Overall, phase 5 models of this design, which involved modifications to optimise the resolution and quality, were found to have the best quality, with the smoothest surfaces, good filament colour distributions, and improved resolutions compared to phases 1–4. Phase 1 remotely-3D-printed model dosage forms had the roughest surfaces to touch compared to phases 2–5 remotely-3D-printed model dosage forms. The remotely-3D-printed model design 2 (phases 1–5), which had an outer torus shape with a flat disc shape inserted into the hollow area achieved by dual extruder FDM printing, is shown in Figure 2B. The phase 1 remotely-printed design 2 model tablets did not have optimal resolutions and well-separated colour distributions. Phases 1 and 2 remotely-printed prototypes had the roughest surfaces upon touching, whereas phase 5 had the smoothest surface among all. Phase 3 to 5 remotely-printed models showed a uniform colour distribution and overall visual appearance.

In general, design 1, 2, and 3 remotely-printed prototypes showed great reproducibility; the mean diameter, length, and thickness had small variations (with a standard deviation of less than 0.8 mm) with the exceptions of design 2–phase 5 and design 3–phase 3 remotely-printed solid dosage form prototypes, which saw larger standard deviation variations at 2.12 and 1.41 mm, respectively. As expected, the overall weight and theoretical volume (mm^3^) increased with an increase in prototype dimensions. Images from Figure 2A–D were used to measure the 2D surface areas of the remotely-printed solid dosage form prototypes. The 2D surface area was expected to have a much smaller surface area compared to the theoretical surface area. Results shown in Table 3 support this statement; for example, the design 1–phase 1 remotely-printed tablet 2D surface area (using Figure 2A) was 1.38 mm^2^, and 298.45 mm^2^ when calculated theoretically.

### 3.3. Design 4—Gummy Bear Shape

Images of remotely-printed prototypes for design 4 (i.e., 3D-printed gummy bear shape prototype solid dosage forms) phases 1 to 5, and the physical properties, are shown in Figure 3 and Table 4, respectively. The “belly” of the remotely printed design (in phases 1 and 2) showed evidence of the “staircase effect” where layering or layered marks were visible on 3D-printed parts, resulting in a rougher feel of the prototype. The “face” features of the designs were not distinguishable in remotely-printed prototypes from phases 1 and 2. The staircase effect was reduced in remotely-printed phase 2 to 5 designs with an increase in appearance, smoothness to touch, and overall improved quality. The feel of a solid dosage form greatly influences patient acceptability [19]. The theoretical surface area and volume were calculated using the formula for calculating a rectangular because the CAD was developed from a rectangular shape before further modifying into a gummy bear shape; therefore, the values were expected to be slightly greater. As a result of this, the values will be overestimated in the facial parts of the gummy bears as they were made up of irregular shapes.

### 3.4. Design 4—Different Infill Densities

Overall, the focus of the work detailed in this study was to explore the remote printability of simple and complex pharmaceutically relevant designs. Considerations of 3D printing in pharmaceutical sciences include varying the percentage infill density (also referred to as the infill percentage) as a strategy to generate chewable and more flexible solid dosage forms. Such formulations are ideal for patients with swallowing difficulties. To push the potential of remote 3D printing of solid dosage forms (prototypes) in this study using the DEFEND3D platform, the infill densities of design 4, as well as the phase 3 prototypes, were remotely 3D-printed at 0, 15, 50, and 100% infill densities (Figure 4). The flexibility (from a patient perspective) was explored by bending each resultant remotely-printed product by hand, with flexibility increasing with decreased infill densities, as shown in Figure 4. The infill density influenced the overall weight of the remotely-printed prototype (as expected), where 100% infill density had the greatest weight (i.e., 1.16 g) compared to the tablet with 0% infill density (0.58 g) (Table 5). This is because the greater the infill percentage, the more polymer is deposited inside the object, resulting in a lesser deformation [28,29]

## 4. Discussion

The proof-of-concept study detailed here addressed the potential of secure (using the SSTP, VICI-patented DEFEND3D platform) remote 3D (FDM) printing of simple and complex pharmaceutically relevant designs as it related to patient experiences when taking and accepting their medications, CAD, and printing optimisation. Solid dosage form prototypes were generated using model polymers, TPU and PLA. All simple and complex designs were successfully remotely and securely 3D-printed (FDM) using the DEFEND3D platform. For all designs, phase 5 models through the DEFEND3D profile (which involved modifications to optimise resolution and quality) were found to have the best quality, smoothest surfaces, good filament colour distributions, and improved resolutions compared to phases 1–4 of all designs. Physical properties (i.e., diameter, length, thickness, weight, surface area, and volume (both theoretical and experimental)) increased with increased prototype dimensions, as expected. Further work was explored with design 4 with remote 3D printing of prototypes with varied infill densities. Varying the infill density in the development of 3D-printed solid dosage forms expands the application of the resultant products as chewable and more flexible dosage forms. Design 4–phase 3 prototypes at 0, 15, 50, and 100% infill densities were successfully remotely 3D-printed. Flexibility (from a patient perspective) was greatest at the lowest infill density (i.e., 0% infill density %).

This work highlights the potential of secure remote 3D (FDM) printing of prototype solid dosage forms resulting in products with good reproducibility, resolution, and quality towards advancement in telemedicine and digital pharmacies. The ability to provide a healthcare service that would start with a consultation, diagnosis, a prescription, and ideally dispensing of the appropriate medicinal product remotely [2], will advance the potential of telemedicine to wider populations and regions globally. This has the potential to reduce global medicine access issues. The uptake of this emerging healthcare process requires barriers to be addressed to facilitate its advancement.

Medication manufacturing and dispensing as it relates to telemedicine and digital pharmacies can be supported by the implementation of 3D printing in the telemedicine care cycle. However, barriers to uptake need to be addressed. The cybersecurity risk associated with the remote digital transfer of a CAD file (acting as the electronic prescription to the printer) has been explored in this study with the use of the DEFEND3D platform, a patented SSTP, VICI designed to enhance the cybersecurity of remote 3D-printed products. The DEFEND3D pre-defines each printer’s profile by selecting the appropriate print speed at various points, layer height, and infill percentage to ensure the optimised quality of prints to be produced [12]. DEFEND3D’s commercial application allows the functionality to drag and drop CAD files into an application within a trusted environment without any knowledge of slicing software and with no 3D printing experience. These files are then sliced for use in several integrated FDM-type desktop machines that have been pre-defined by a DEFEND3D CAD engineer to allow an optimised print performance. This could be advantageous in digital pharmacies to ensure consistency across all prints, making sure that accurate doses are present in each formulation, as well as reducing the labour burden. Various regulatory concerns are circulating regarding the introduction of 3D printing into pharmacies. Copyright issues are often encountered in 3D printing. The CAD designed using dedicated 3D software by pharmacists undoubtedly involves human intellect, which is considered an intellectual property that needs to be protected against proliferation use [30]. DEFEND3D allows the secure transmission of virtual inventory to be delivered instantly without revealing intellectual property. The 3D file will always remain on the source computer, meaning the file cannot be stolen or manipulated by someone else.

## 5. Conclusions

Our work has confirmed the ability of the platform to successfully remotely 3D-print simple and complex pharmaceutically relevant designs at various infill densities. The limitation of the work presented here involves the use of model polymers and not pharmaceutically relevant polymers. This study focused on remote printability as it related to the shape complexity of pharmaceutical relevance and not the materials used. TPU and PLA were chosen due to their flexibility, ease of printing via FDM 3D printing, and to prevent breakage printing. This study has confirmed the possibility of secure remote printing of pharmaceutically relevant-shaped solid dosage forms.

Further work could be conducted using the same designs chosen in this study but with pharmaceutically relevant polymers used in hot-melt extrusion (HME) with demonstrated suitability for FDM 3D printing [31], such as poly(vinyl alcohol) [32]. However, it should be noted that any challenges that may occur with pharmaceutically relevant polymers are likely to be related to the polymer’s printability and printer choice as opposed to the ability of the CAD file to be transferred to the printer remotely.

## Figures and Tables

**Table 1:** Summary of printing phases, polymers used, printer type (i.e., single or dual extruder), and printing parameters.

	Polymer	Printer Type i.e., Single/Dual Extruder	Printing Parameters			
			Nozzle Extrusion Temperature °C	Base Speed mm/s	Layer Height mm	Infill Density %
Phase 1	TPU	Single extruder	220	40	0.2 60	60
Phase 2			210	35		30
Phase 3				20	0.1	0, 15, 50, 100
Phase 4	PLA	Dual extruder		50		15
Phase 5						

**Table 2:** Equations used to calculate the theoretical surface area (SA) and volume (Vol).

Equation	Equation Number
$\mathrm{SA}\;\mathrm{of}\;\mathrm{design}\;1=2\pi r^{2}+2\pi rt$	(1)
$\mathrm{SA}\;\mathrm{of}\;\mathrm{design}\;2=2\pi r^{2}+2\pi rt$	(2)
$\mathrm{SA}\;\mathrm{of}\;\mathrm{design}\;3=\left[2\pi r_{1}^{2}+2\pi r_{1}t\right]-\left[2\pi r_{2}^{2}+2\pi r_{2}t\right]$	(3)
SA of design 4=2dt+2dl+2tl	(4)
$\mathrm{Vol}\;\mathrm{of}\;\mathrm{design}\;1=\pi r^{2}t$	(5)
$\mathrm{Vol}\;\mathrm{of}\;\mathrm{design}\;2=\pi r^{2}t$	(6)
$\mathrm{Vol}\;\mathrm{of}\;\mathrm{design}\;3=\left[\pi r_{1}^{2}t\right]-\left[\pi r_{2}^{2}t\right]$	(7)
Vol of design 4=d×l×t	(8)

**Table 3:** Physical properties of remotely-3D-printed model designs 1 to 3. Data for diameter, length, and thickness represent the mean ± standard deviation.

	Diameter ± SD (mm)	Length ± SD (mm)	Thickness ± SD (mm)	Weight (g)	2D Surface Area (mm^2^)	Theoretical Surface Area (mm^2^)	Theoretical Volume (mm^3^)
**Design 1**							
Phase 1	10.00 ± 0.71	10.00 ± 0.71	4.50 ± 0.00	0.28	1.38	298.45	353.43
Phase 2	10.00 ± 0.00	10.00 ± 0.00	4.50 ± 0.07	0.26	1.35	298.45	353.43
Phase 3	16.00 ± 0.00	16.00 ± 0.71	6.00 ± 0.71	0.67	5.53	703.72	1206.37
Phase 4	15.00 ± 0.00	15.00 ± 0.00	6.00 ± 0.00	0.85	4.11	636.17	1060.29
Phase 5	6.00 ± 0.00	6.00 ± 0.00	3.00 ± 0.00	0.09	0.68	113.10	84.82
**Design 2**							
Phase 1	12.00 ± 0.71	12.00 ± 0.71	3.00 ± 1.41	0.37	1.33	339.29	339.29
Phase 2	12.00 ± 0.71	12.00 ± 0.71	3.00 ± 1.41	0.43	2.27	339.39	339.39
Phase 3	15.00 ± 0.71	15.00 ± 0.71	6.00 ± 1.41	0.89	4.37	636.17	1060.29
Phase 4	14.00 ± 0.00	14.00 ± 0.00	5.00 ± 0.71	0.81	4.49	527.79	769.69
Phase 5	8.00 ± 2.12	8.00 ± 2.12	3.00 ± 0.71	0.11	0.77	175.93	150.80
**Design 3**							
Phase 1	11.00 ± 0.00	11.00 ± 0.00	6.00 ± 0.71	0.26	2.21	1.40	146.08
Phase 2	11.00 ± 0.00	11.00 ± 0.00	5.00 ± 0.00	0.33	1.62	1.37	175.93
Phase 3	13.00 ± 1.41	16.00 ± 0.71	6.00 ± 0.71	0.49	3.31	2.56	164.93
Phase 4	13.00 ± 0.71	16.00 ± 0.00	5.00 ± 0.71	0.43	3.52	2.19	155.50
Phase 5	8.00 ± 0.00	10.00 ± 0.00	5.00 ± 0.71	0.14	1.61	1.41	75.40

**Table 4:** Physical properties of 3D-printed gummy bear shape tablet (design 4). Data for diameter, length, and thickness represent the mean ± standard deviation (SD), where *n* = 2.

DESIGN 4	Diameter (mm)	Length (mm)	Thickness (mm)	Weight (g)	2D Surface Area (mm^2^)	Theoretical Surface Area (mm^2^)	Theoretical Volume (mm^3^)
Phase 1	15.00 ± 0.00	25.00 ± 0.71	2.00 ± 0.71	0.67	3.16	910.00	750.00
Phase 2	15.00 ± 0.00	26.00 ± 0.00	4.00 ± 0.00	0.60	4.27	1168.00	1664.00
Phase 3	14.00 ± 0.71	24.00 ± 0.00	3.00 ± 0.35	0.58	3.92	900.00	1008.00
Phase 4	15.00 ± 0.71	24.00 ± 0.00	3.00 ± 0.35	0.97	4.25	954.00	1080.00
Phase 5	10.00 ± 0.35	15.00 ± 0.00	3.00 ± 0.71	0.21	1.84	450.00	450.00

**Table 5:** Different infill densities of 3D-printed gummy bear shape tablets (design 4). Data for diameter, length, and thickness represent the mean ± standard deviation (SD), where *n* = 2.

Infill Densities	Diameter (mm)	Length (mm)	Thickness (mm)	Weight (g)	2D Surface Area (mm^2^)	Theoretical Surface Area (mm^2^)	Theoretical Volume (mm^3^)
0%	14.00 ± 0.71	24.00 ± 0.00	3.00 ± 0.35	0.58	3.92	900.00	1008.00
15%	14.00 ± 0.71	24.00 ± 0.71	3.00 ± 0.00	0.62	2.69	900.00	1008.00
50%	13.00 ± 0.00	24.00 ± 0.00	3.00 ± 0.00	0.67	3.20	846.00	936.00
100%	14.00 ± 0.00	25.00 ± 0.71	3.00 ± 0.35	1.16	3.65	934.00	1050.00

## Data Availability

Data available on request.
